# A vermal Purkinje cell simple spike population response encodes the changes in eye movement kinematics due to smooth pursuit adaptation

**DOI:** 10.3389/fnsys.2013.00003

**Published:** 2013-03-13

**Authors:** Suryadeep Dash, Peter W. Dicke, Peter Thier

**Affiliations:** Department of Cognitive Neurology, Hertie Institute for Clinical Brain Research, University of TübingenTübingen, Germany

**Keywords:** smooth pursuit eye movements, cerebellar cortex, Purkinje cells, vermis, monkey

## Abstract

Smooth pursuit adaptation (SPA) is an example of cerebellum-dependent motor learning that depends on the integrity of the oculomotor vermis (OMV). In an attempt to unveil the neuronal basis of the role of the OMV in SPA, we recorded Purkinje cell simple spikes (PC SS) of trained monkeys. Individual PC SS exhibited specific changes of their discharge patterns during the course of SPA. However, these individual changes did not provide a reliable explanation of the behavioral changes. On the other hand, the population response of PC SS perfectly reflected the changes resulting from adaptation. Population vector was calculated using all cells recorded independent of their location. A population code conveying the behavioral changes is in full accordance with the anatomical convergence of PC axons on target neurons in the cerebellar nuclei. Its computational advantage is the ease with which it can be adjusted to the needs of the behavior by changing the contribution of individual PC SS based on error feedback.

## Introduction

Ever since the seminal paper of Marr (1969), the cerebellum has been implicated in motor learning, here broadly understood as the ability to optimize motor acts based on feedback on previous realizations. Evidence in support of this role has come from studies of a number of motor tasks including both non-associative motor learning like saccadic adaptation or adaptation of the vestibulo-ocular reflex as well as associative learning like the conditioning of the nictitating membrane reflex (McCormick et al., 1982; Yeo et al., 1985; Barash et al., 1999; Rambold et al., 2002; Blazquez et al., 2003). Yet, the computational principle underlying the cerebellar role in motor adaptation has remained contentious as exemplified by the long-lasting and continuing discussion of the relationship between cerebellum-based motor learning as studied on a behavioral level and heterosynaptic long term depression (LTD) of parallel fiber synapses on Purkinje cells (PCs) (Ito, 2006). Any attempt to unravel the computational principle underlying cerebellum based motor learning requires an understanding of the output code used to convey the consequences of the learning to the structures in cerebral cortex and elsewhere, responsible for the planning and execution of the behavior. The only axons leaving cerebellar cortex are those of PCs. Hence, the vehicle carrying the code must be the action potentials generated by these neurons. PCs exhibit two distinct types of spikes. The complex spike (CS) is fired at conspicuously low frequencies and is fully determined by the climbing fiber afferents contacting PCs. The simple spike (SS) is influenced by both the mossy fiber afferents as well as by cerebellar interneurons and usually exhibits significant spontaneous activity. It is well accepted that SS, whose firing rates are about two orders of magnitude higher than those of the CS, can have a significant impact on the nuclear target neurons (Monsivais et al., 2005; also see Hoebeek et al., 2010 and Blenkinsop and Lang, 2011). This is why any contribution of the cerebellar cortex to motor learning must have a major contribution by the SS. Indeed, previous work on various forms of cerebellum-dependent motor learning has demonstrated learning related changes in SS firing of individual SS units (Ojakangas and Ebner, 1992; Kahlon and Lisberger, 2000; Blazquez et al., 2003; Catz et al., 2008; Medina and Lisberger, 2009). Yet, the associations between SS firing of single PCs and changes of behavior have been weak in most of the studies. This is arguably an inevitable consequence of the high degree of anatomical convergence of individual PC axons on neurons in the deep cerebellar nuclei (Palkovits et al., 1977; see also Person and Raman, 2012 for physiological data suggesting a more modest convergence). If this interpretation were correct one would expect to see that the collective SS activity of the PCs converging on an individual target neuron should provide a much better description. Actually, we recently demonstrated that a SS population vector based on recordings of PC SS from the oculomotor vermis (OMV) approximating this collective input is able to accurately describe the kinematics of two very dissimilar types of visually guided eye movements, saccades and smooth pursuit (Thier et al., 2000; Dash et al., 2012). Lesion experiments have already established a role of OMV in the adaptation of both saccade amplitude (Takagi et al., 1998; Barash et al., 1999) and initial pursuit velocity (Takagi et al., 2000). Here we sought support for the hypothesis that an adjustment of this population code could account for motor learning by using smooth pursuit eye movements (SPEM) as a model system.

## Materials and methods

### Animal and surgical procedures

Two male rhesus monkeys (*Macaca mulata*) (A and N) were used in this study. They were prepared for eye position recording and extracellular single unit recording using procedures explained elsewhere (Dash et al., 2012). All procedures complied with the NIH Guide for Care and Use of Laboratory Animals and were approved by the local animal care committee.

### Behavioral paradigms

The monkeys were trained to elicit SPEM in two horizontal directions (0 and 180°) in response to a step-ramp sequence of target motion. The target motion consisted of an initial target step away from the central fixation point in a direction opposite to the direction of the subsequent target ramp. The step amplitude depended on the ramp velocity and the pursuit latency of the individual monkey and was chosen such as to have the target back at straight ahead at pursuit onset, thereby minimizing the need for catch-up saccades (Rashbass, 1961). Pursuit onset was determined by identifying the point in time at which eye velocity exceeded two times the standard deviation of eye velocity during fixation of the stationary target (=baseline eye velocity).

**Figure 1:**
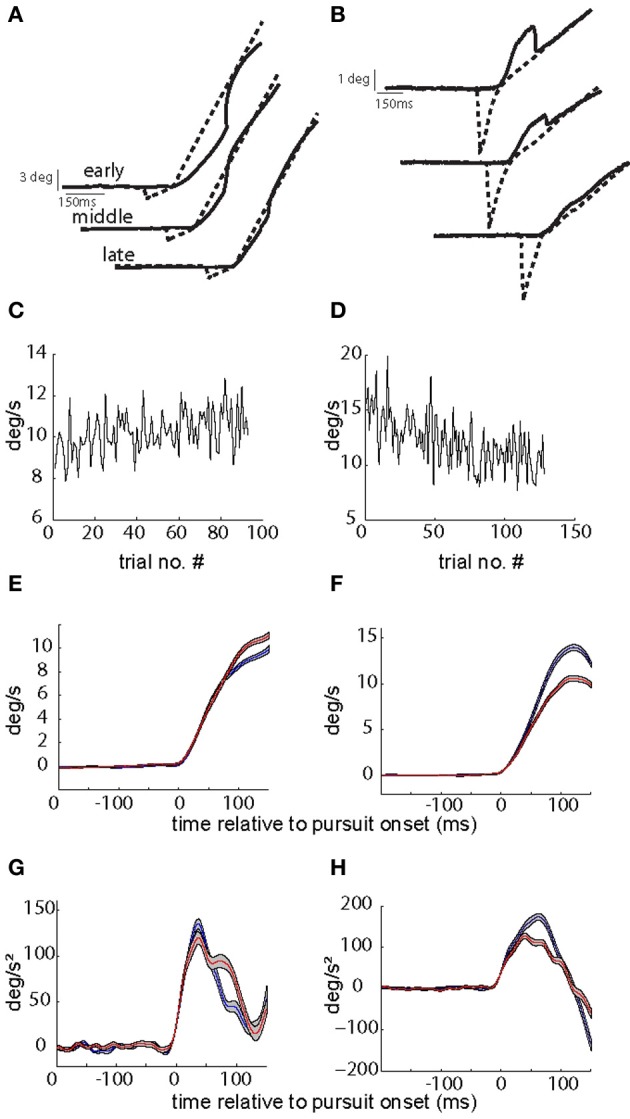
**Kinematic changes associated with gain-increase (A,C,E,G) and gain-decrease SPA (B,D,F,H). (A,B)** Show exemplary target and eye position traces from early, middle, and late in the session during gain-increase SPA **(A)** and gain-increase SPA **(B)**. **(C,D)** Plot peak eye velocity (in the first 150 ms after SPEM onset) as function of trial number during the course of SPA. Peak velocity increases during the course of gain-increase SPA **(C)** and decreases during gain-decrease SPA **(D)**. **(E,F)** Show the average velocity profiles (with standard errors) in the first (blue) and last (red) quarters of adaptation sessions leading to gain-increase SPA **(E)** or gain-decrease SPA **(F)**. Note that that peak velocity is not attained in the first 100 ms during gain-increase SPA. **(G,H)** Depict the average acceleration profiles (with standard errors) in the first (blue) and last (red) quarters of adaptation sessions leading to gain-increase SPA **(G)** or gain-decrease SPA **(H)**. While gain-increase SPA is brought about by prolonging the acceleration duration, a drop in peak acceleration is responsible for gain-decrease SPA.

Two behavioral paradigms were used to increase or decrease the gain of initial SPEM respectively [gain increase vs. decrease smooth pursuit adaptation (SPA)]. SPA was induced by introducing a change in target velocity around the time the eye movement was expected to commence, i.e., at the pre-determined pursuit latency. The pursuit-related discharge in our previous study (Dash et al., 2012) was aligned with movement onset, rather than target onset, ruling out any substantial visual response. Hence, we used a step-ramp paradigm with non-blanked target ramp. In different experiments, target velocity was either increased in each trial of a given adaptation experiment (from 5 to 15°/s; “gain-increase SPA”) or decreased (from 15 to 5°/s; “gain-decrease SPA”). The number of trials per adaptation session varied between 80–200 trials (median = 120; mean = 124; standard deviation = 30). For every trial we calculated the maximum eye velocity in the first 150 ms of eye movement and for each experiment we compared the average of the maximum eye velocity for the first quarter with the average for the last quarter of the trials. We determined the maximum eye velocity in the first 150 ms rather than in the first 100 ms as done in previous studies of SPA (Kahlon and Lisberger, 1996, 2000) as in our material, peak velocity was rarely attained within the first 100 ms but in many cases required up to 150 ms to be achieved. SPA was considered significant if the averages were significantly different (*t*-test, *p*-value < 0.05), and only those sessions were considered for further analysis. Figure 1 shows kinematic changes associated with exemplary sessions of gain-increase and gain-decrease SPA. Across all the sessions average percentage change in gain (% change in peak velocity between first and last quarter) was 11.3 and 11.6% for gain-increase and gain-decrease SPA, respectively.

### Electrophysiological procedures

An anatomical MRI (magnetic resonance imaging) obtained after the implantation of the recording chamber was the basis for the initial approach of the electrodes to the OMV. Additional clues were provided by the characteristic features of the signals picked up by the microelectrode entering the OMV, namely the dramatic increase in background noise and the presence of saccade related background activity originating from granule cells as well as the abundance of saccade related PC SS.

Recordings from the OMV were carried out using a multi-electrode positioning system with four linearly aligned (spacing = 1 mm) and independently positioned glass-coated tungsten microelectrodes (Alpha Omega Engineering; impedance <1.2 megaohms). After appropriate amplification and filtering, individual spikes were separated online based on template matching as provided by the Alpha-Omega Engineering Multi Spike Detector (for a detailed description of neuron isolation, see Catz et al., 2005). Classification of these units as PC SS was based on the occurrence of CS with consecutive pauses in SS discharge and consideration of their high level of spontaneous SS activity. All the neurons in our sample exhibited spontaneous SS discharge rates as measured during fixation of a stationary target of more than 30 spikes/s, with the maximum spontaneous firing being as high as 200 spikes/s. No other cell type in the cerebellar cortex exhibits such high spontaneous firing levels (Armstrong and Rawson, 1979; Prsa et al., 2009). A PC was considered pursuit related if its activity during the first 150 ms of eye movement was significantly different from the preceding baseline (200 ms during fixation period) (Wilcoxon rank test; *p* < 0.05), for at least one of the two horizontal directions (0 and 180°).

### Data analysis

All analyses were performed offline using customized MATLAB programs (MATLAB, The MathsWorks Inc., MA). Instantaneous eye velocity and acceleration were derived from the eye position records, which were sampled at a rate of 1 kHz. The horizontal and vertical eye position records were smoothed using a Savitzky–Golay filter (window = 20 samples; polynomial degree = 4), which replaces the data points in the specified window by a polynomial fit of chosen degree and subsequently differentiated (Savitzky and Golay, 1964). Trials with saccades during the initial 150 ms of eye movement were discarded. Saccades were detected using a velocity threshold of 50°/s. Trials with a pursuit latency of more than 250 ms were deemed to reflect an un-attentive state of the monkey, which is why such trials were as well discarded. We estimated the instantaneous firing rate of the recorded neurons with a continuous spike density function, generated by convoluting the spike train with a Gaussian function of σ = 10 ms width. We converted the discharge into spike density functions in order to obtain the continuous description of neuronal activity needed for the investigation of the relationship between kinematic variables and neuronal activity.

### Single unit analysis

SPA sessions were confined to the horizontal pursuit direction. A given PC was considered if it displayed a significant response to horizontal pursuit. In other words, no attempt was made to determine the preferred direction of SPA in order to later test for SPA related changes in this particular direction. All the subsequent behavioral and electrophysiological measurements were done only for the direction chosen for adaptation. We preferred tests of horizontal smooth pursuit to expedite the collection of a sufficiently large sample of consistent behavioral data allowing us to test for SPA related discharge changes. The choice of the direction of adaptation (gain-increase or gain-decrease) was made randomly.

The average discharge rate in the first 150 ms after pursuit onset was taken as “*test period*” and the average discharge rate in the 200 ms preceding target movement onset was considered as “*baseline period*.” We computed the coefficient of modulation by SPA (*C*_adapt_) for each PC SS using the following equation:

(1) $$C_{\text{adapt}}=(c\text{/}d-a\text{/}b)/(c\text{/}d\text{+}a\text{/}b)$$

where *a* is the average discharge rate in the test period of the first quarter of trials, *b* the average in the baseline period of the first quarter of trials, *c* the average discharge rate in the test period of the last quarter of trials and *d* the average activity during baseline period of the last quarter of trials. *C*_adapt_ will be zero in case adaptation has no influence on the response, it will lie between 0 and 1 in the case of an adaptation-induced increase in discharge and it will lie between 0 and −1 if adaptation led to a decrease in discharge. An index of pursuit modulation (*C*_gain_) for ascertaining if the pursuit related response was excitatory or inhibitory was obtained by dividing the average activity during the test period based on the pre-adaptation trials (target velocity = 10°/s) by the average activity during the baseline period for the same trials. PC SS showing an excitatory pursuit response will have a *C*_gain_ above 1 while those showing inhibitory pursuit responses will have a *C*_gain_ below 1.

### Population spike density function

We calculated a population spike density function (later simply referred to as *population response or population signal*) by estimating the instantaneous firing rate of every single PC with a continuous spike density function, obtained by convoluting spike trains with a Gaussian function of σ = 10 ms width, and thereafter averaging the individual spike density functions (Silverman, 1986). In order to identify changes of the population spike density function across an adaptation session, we divided every adaptation session into four bins comprising equal numbers of trials and generated individual population average spike density functions for each of the four bins.

### Multiple linear regression analysis of discharge patterns

In order to quantify the relationship between neuronal activity and eye movement kinematics we modeled the discharge rate spk(*t*) as a linear combination of eye position [pos(*t*)], eye velocity [vel(*t*)], and eye acceleration [acc(*t*)]:

(2) spk(t−Δ)=e×pos(t)+f×vel(t)+g×acc(t)+h

The four coefficients *e*, *f*, *g*, *h* and Δ were estimated to minimize the deviation between the reconstructed discharge rate and the actual discharge rate as indicated by a maximal coefficient of determination (*CD* = *r*^2^) for the equation (Shidara et al., 1993). The CD value corresponds to 1 minus the ratio of the error sum of squares (sse) and the total sum of squares (sst). The sse is the square of the difference between the reconstructed PC firing frequency and the observed firing frequency, and sst is the square of the difference between the observed PC SS firing frequency and the temporally averaged firing frequency of the PC SS for the whole duration (a constant value). The CD value ranges between 0 and 1. The time period from 100 ms before SPEM onset to 150 ms after SPEM onset was used for the estimation of the coefficients. A Student's *t*-test was applied to test the null hypothesis that the coefficient for each eye movement parameter is equal to zero. *P*-values less than 0.05 indicate that a given coefficient is significantly different from zero.

We used fixed values of Δ ranging between −20 and 20 ms in increments of 5 ms (total nine fixed Δ). As will become clear in the result section, we studied the changes of CD as well as of kinematic coefficients (*e*, *f*, and *g*) during the course of SPA. A fixed Δ and a wide range of Δ′s allowed a simpler comparison as well as a detailed account of temporal relationship of spiking activity with the movement kinematics. Overall, a Δ of 10 or 15 ms (spikes leading the movement) yielded the best fits. Therefore all the figure illustrations are shown at Δ = 15.

## Results

We recorded pursuit-related SS of 163 PCs (PC SS units, for the sake of simplicity referred to as PC SS) from the OMV of two rhesus monkeys (monkey A: 42 PC SS, monkey N: 121 PC SS), which could be followed during either gain-increase (*n* = 104) or gain-decrease SPA (*n* = 59). In all cases SPA led to significant changes in eye velocity in the direction tested. Out of 104 PC SS tested for gain-increase SPA, 73 PC SS exhibited excitatory pursuit related responses (*C*_gain_ > 1) and the remaining 31 PC SS showed inhibitory pursuit related responses (*C*_gain_ < 1). Similarly, 50 of the 59 PC SS tested for gain-decrease SPA exhibited excitatory pursuit related responses, while the remaining nine PC SS showed inhibitory pursuit-related activity.

**Figure 2:**
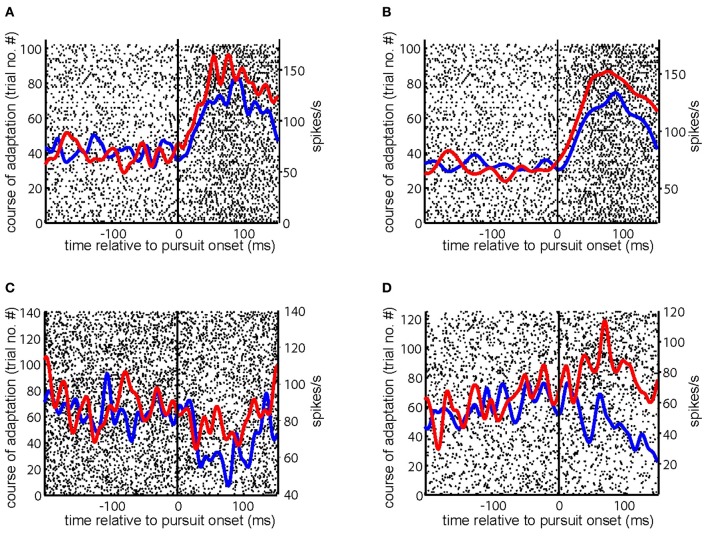
**Responses of exemplary PC SS during gain-increase SPA. (A–D)** Show the raster plot of a PC SS during the course of gain-increase SPA with superimposed spike density functions for the first (blue) and the last (red) quarter of the trials. The solid vertical line indicates SPEM onset. The course of adaptation is from bottom to top.

**Figure 3:**
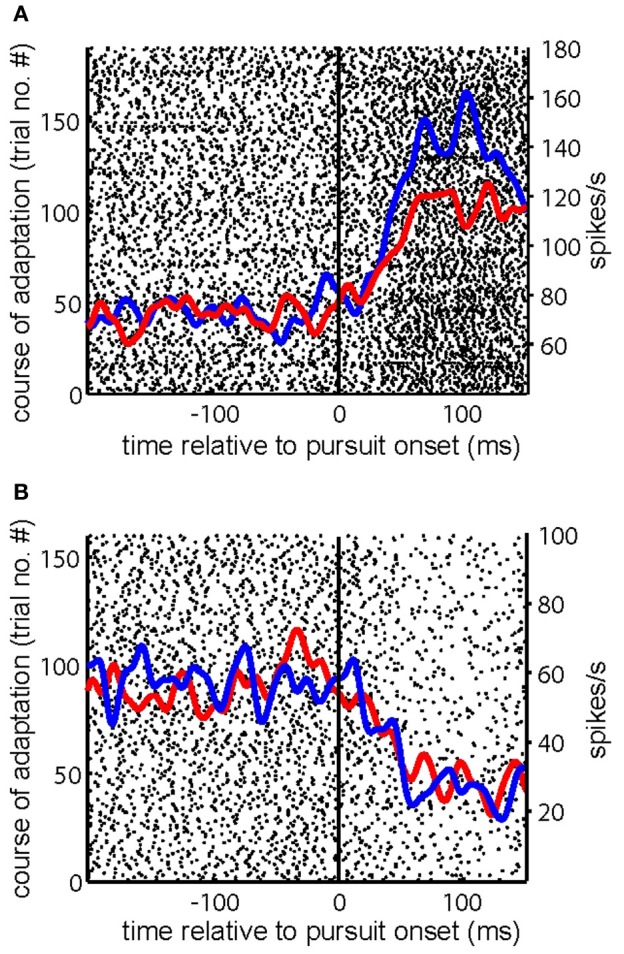
**Examples of PC SS during gain decrease SPA. (A,B)** Show the raster plot of a PC SS during the course of gain-decrease SPA with superimposed spike density functions for the first (blue) and the last (red) quarter of the trials. The solid vertical line indicates SPEM onset. The course of adaptation is from bottom to top.

Many (*n* = 69) of the 104 PC SS studied during gain-increase SPA demonstrated an increase of their pursuit related discharge from the first to the last quarter of trials (*C*_adapt_ > 0) of an adaptation session. Figures 2A–D present exemplary PC SS exhibiting increase of its firing rate during gain-increase SPA (*C*_adapt_ > 0.1). Similarly, many (*n* = 40) of the 59 PC SS tested for gain-decrease SPA showed a decrease in firing rate between the first and the last quarter of trials (*C*_adapt_ < 0). Figure 3A depicts an exemplary PC SS demonstrating a decrease of its firing rate during gain-decrease SPA (*C*_adapt_ < −0.1). Figure 3B is an exemplary PC SS with inhibitory pursuit response showing no change in firing during gain-decrease SPA.

**Figure 4:**
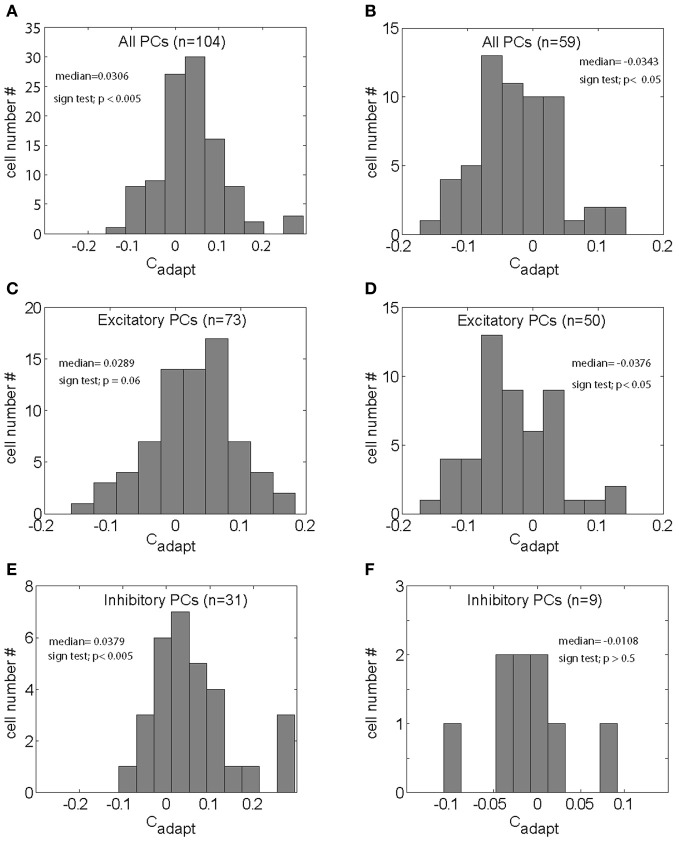
**(A,B)** Distributions of the index capturing the adaptation related modulation (*C*_adapt_) for all the PC SS tested for gain-increase SPA **(A)** and gain-decrease SPA respectively **(B)**. **(C,D)** Distributions *C*_adapt_ for all excitatory PC SS tested for gain-increase SPA **(C)** and gain-decrease SPA **(D)**, respectively. **(E,F)** Distribution of *C*_adapt_ for the inhibitory PC SS subjected to gain-increase **(E)** and gain-decrease SPA respectively **(F)**.

In an attempt to capture the influence of SPA on the discharge at the population level, we analyzed the distributions of *C*_adapt_ for gain increase (Figure 4A) and for gain decrease SPA (Figure 4B). Most PC SS subjected to either form of SPA exhibited non-zero values of *C*_adapt_, reflecting an impact of SPA on the discharge. Actually, the distribution of *C*_adapt_ for gain-increase SPA was significantly shifted away from zero toward positive values (Figure 4A; *p* < 0.005, sign test), reflecting an increase of the pursuit-related discharge. Conversely, the *C*_adapt_ distribution for gain-decrease SPA was significantly shifted away from zero toward negative values (Figure 4B; *p* < 0.05, sign test), indicating a decrease of the pursuit-related discharge.

**Figure 5:**
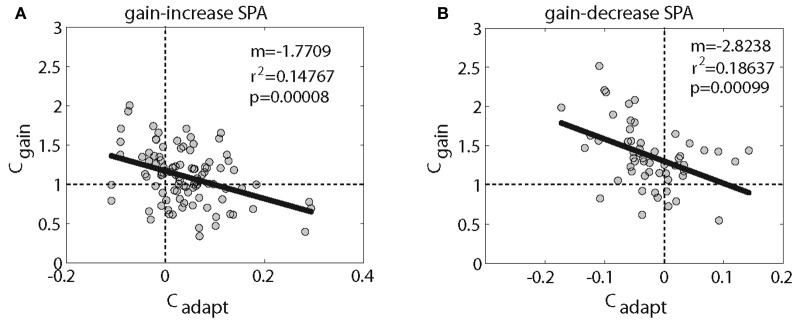
**Shows the relationship between *C*_gain_ (excitatory PC > 1; and inhibitory PC < 1) and *C*_adapt_ (adaptation related changes for gain-increase SPA (A) and gain-decrease SPA (B).** A linear regression shows a significant relationship.

We next asked if the change of *C*_adapt_ was different for PC SS with inhibitory and those with excitatory responses, for both gain-increase and gain-decrease SPA. In other words, we wanted to know if the direction of the SPA related changes was determined by the type of SPA or by the pursuit-related response being inhibitory or excitatory. Figure 4C shows the distribution of *C*_adapt_ for excitatory PC SS during gain-increase SPA. It was clearly shifted away from zero toward positive values; however, the shift was not significant (*p* = 0.06, sign test). On the other hand, the distribution of *C*_adapt_ for excitatory PC SS during gain-decrease SPA was shifted significantly away from zero toward negative values (Figure 4D; *p* < 0.05, sign test). The PC SS with inhibitory responses showed a different behavior. During gain-increase SPA we observed a significant shift in the distribution of *C*_adapt_ for inhibitory PC SS away from zero toward positive values (Figure 4E; *p* < 0.005, sign test). In fact, the positive deviation away from zero was much more evident in inhibitory PC SS as compared to the excitatory ones. No shift was observed in the distribution of *C*_adapt_ for inhibitory PC SS during gain-decrease SPA (Figure 4F; *p* > 0.5, sign test). In others words, excitatory responses of PC SS grew in most cases during gain-increase SPA, whereas inhibitory responses got weaker. On the other hand, excitatory responses of PC SS subjected to gain-decrease SPA typically got weaker, whereas inhibitory responses showed no change. To get further insight into relative contribution of excitatory and inhibitory pursuit related PC SS during either gain-increase or gain-decrease SPA, we plotted *C*_gain_ as a function of *C*_adapt_ (Figure 5). Figure 5A shows that inhibitory PCs (*C*_gain_ < 1) exhibited bigger adaptation related changes (*C*_adapt_) during gain-increase SPA (Figure 5A). On the other hand, during gain-decrease SPA, bigger absolute values of *C*_adapt_ were obtained for neurons exhibiting higher *C*_gain_ (Figure 5B).

**Figure 6:**
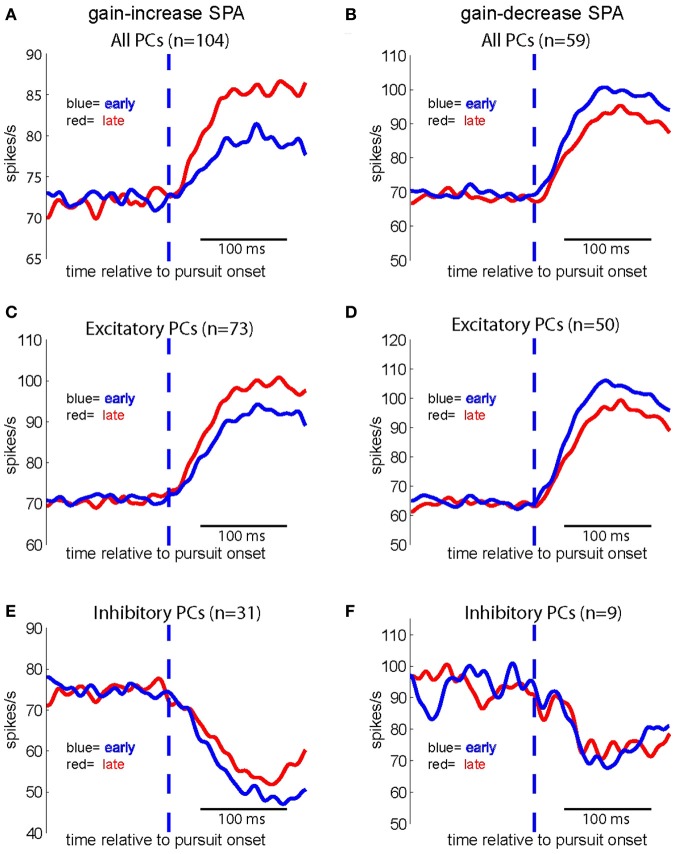
**Population spike density functions for different groups of PC SS subjected to either gain increase (left panels) or to gain decrease SPA (right panels) for the first (red) and the last quarter of trials (blue).** Upper row: population spike density functions based on all PC SS tested for gain-increase SPA (*n* = 104) **(A)** and gain-decrease SPA (*n* = 59) **(B)** middle row: population spike density functions for all excitatory PC SS tested for gain-increase (*n* = 73) **(C)** and gain-decrease SPA (*n* = 50) **(D)** bottom row: population spike density functions for all inhibitory PC SS tested for gain-increase SPA (*n* = 31) **(E)** and gain-decrease SPA (*n* = 9) **(F)**.

Figure 6A shows the population spike density function (“the population response” or “population signal”) based on all neurons (*n* = 104) subjected to gain-increase SPA. The figure compares the population response early during gain-increase SPA (first quarter of trials = blue; mean = 76.36 spikes/s) with the one late during gain-increase SPA (last quarter of trials = red; mean = 83.10 spikes/s). The comparison between the first and the last quarter of trials documents a highly significant increase in the strength of the population signal as a consequence of gain-increase SPA in the test period (first 150 ms after SPEM onset) (Wilcoxon signed rank test, *p* < 0.001), whereas the population discharge in the baseline period (150 ms preceding SPEM onset) did not change (Wilcoxon signed rank test, *p* > 0.05). Accordingly, Figure 6B shows that the population response based on all 59 units tested for gain-decrease SPA exhibited a significant reduction during the test period (Wilcoxon signed rank test, *p* < 0.001; from 93.89 spikes/s in first quarter of trials to 87.11 spikes/s in the last quarter), whereas the population discharge during the baseline period did not change (Wilcoxon signed rank test, *p* > 0.05). When the population response was based on the excitatory PC SS only (*n* = 73), a similar increase in the strength of the population signal, specific to the test period was observed in the case of gain-increase adaptation (Figure 6C; Wilcoxon signed rank test, *p* < 0.001), without any change during the baseline period (Wilcoxon signed rank test, *p* > 0.05). Conversely, the pursuit-related population response based on excitatory PC SS only (*n* = 50) declined during gain decrease SPA as indicated by a significant and specific drop of the population signal in the test period signal (Figure 6D; Wilcoxon signed rank test, *p* < 0.001), without any change during baseline period. The same analysis applied to the population response comprising inhibitory PC SS revealed a highly significant effect for the test period during gain-increase SPA (Figure 6E, *n* = 31, Wilcoxon signed rank test, *p* < 0.005), without any change during the baseline period. However, no significant change was observed for gain-decrease SPA, neither during the test period nor during the baseline period (Figure 6F, Wilcoxon signed rank test, *p* > 0.05, *n* = 9). In summary, both excitatory and inhibitory PC SS showed significant increases of their discharge at the population level during gain-increase SPA, while only excitatory PC SS exhibited a decrease in population firing rate during gain-decrease SPA.

**Figure 7:**
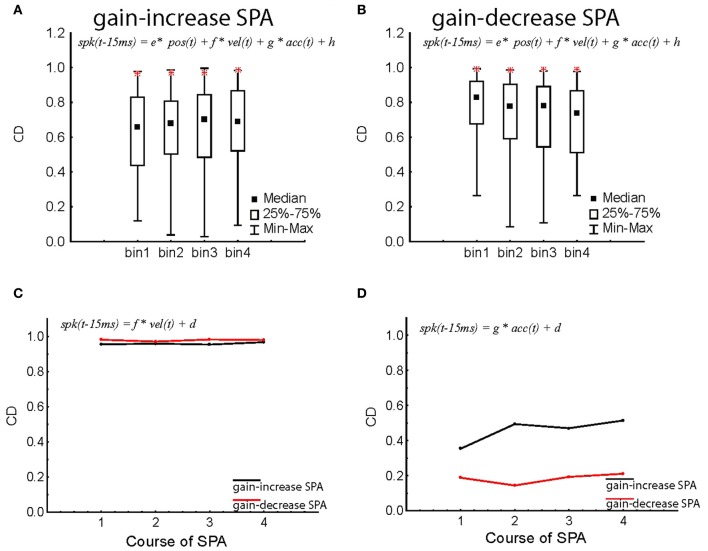
**Changes in the coefficient of determination (*CD* = *r*^2^; see “Materials and Methods,” Equation 2) of individual PC SS and population response during gain-increase SPA (A) and gain-decrease SPA (B).** The distribution of CD for individual PC SS is shown in median/quartile values of CDs throughout the course of SPA divided up into four bins for the entire population of PC SS. The CDs obtained for the instantaneous population response are added as red stars in the corresponding bin characterizing the adaptation course. Note that the CDs for the population response are much larger than the vast majority of individual PC SS. **(C,D)** Degraded regression models restricted to eye velocity **(C)** and eye acceleration **(D)** respectively as function of the four bins characterizing the course of SPA. The population response was based on all PC SS, independent of response type. The CDs of gain-increase SPA are shown in black, the ones for gain-decrease SPA in red. All the CDs computed in this figure are for Δ = 15 ms.

We have previously shown that the linear combination of three kinematic variables, namely, eye velocity, eye acceleration, and eye position allows one to precisely predict the instantaneous discharge of the population signal (Dash et al., 2012). We therefore asked if the tight relationship between the population signal and the kinematic variables is maintained during SPA. Maintenance of this tight relationship would suggest that changes of the population signal might actually be responsible for the behavioral changes observed. In order to address this question we subjected the discharge of individual PC SS as well as the population signal to a multiple linear regression analysis with the three kinematic variables eye position, velocity, and acceleration (see “Materials and Methods” for details). The coefficient of determination (*CD* = *r*^2^), reflecting the goodness of the fit, was measured for each of the four bins of trials representing the course of SPA. For both types of adaptation and independent of the period of adaptation considered, the distributions of CD encompassed a wide range of CDs from 0.2 to as high 0.98, indicating that the discharge of individual units may depend very differently on eye movement kinematics. In comparison, the multiple regression analysis of the instantaneous population signal showed a perfect CD close to unity (>0.98) independent of the specific bin reflecting the course of SPA considered. As shown in Figures 7A,B, the population response CDs stayed higher than any individual PC SS throughout the course of both gain-increase and gain-decrease SPA. It is worth pointing out that our sample population activity suffered from a sampling bias with the ratio of excitatory and inhibitory neurons for the gain-increase SPA experiments being 73:31 (~2:1) and that for gain-decrease SPA experiment being 50:9 (~5:1). Therefore, all the results are shown not only for the entire population but also for the sub-populations of excitatory and inhibitory neurons, separately.

Since the kinematic variables defining the eye movement are not independent of each other, we also ran degraded linear regression analyses in which discharge rate was led back to the individual kinematic parameters position, velocity, or acceleration. We also subjected the instantaneous population response to degraded regression analyses, focussing on individual kinematic variables. As clearly shown by Figure 7C, eye velocity was able to explain most of the discharge variance (*CD* > 0.96), independent of the type of SPA and the respective SPA bin. When compared to the full model (Figures 7A,B), the degraded model relying on only the velocity term was almost as good as the full model in explaining the variance of the population response. Eye acceleration was the only kinematic parameter that showed a difference between gain-increase and gain-decrease SPA. While the amount of discharge variance explanation (CD) was on the order of 0.4–0.6 during gain-increase SPA, it was less than 0.2 during gain-decrease SPA (Figure 7D). The correlation with eye position was substantially weaker as reflected by CDs between 0.6 and 0.7, again independent of the type of SPA or SPA bin.

**Figure 8:**
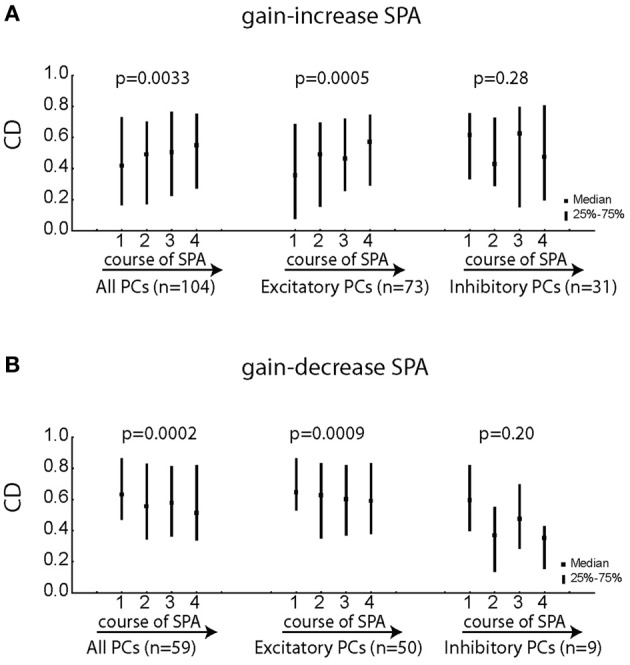
**Changes in the coefficient of determination (*CD* = *r*^2^) for the equation [spk(*t* − Δ) = *f* × vel(*t*) + *h*] with Δ = 15 ms for various subgroups of PC SS during gain-increase SPA (A) and gain-decrease SPA (B).** The left column shows the median/quartile values of CDs for the four bins capturing the course of SPA for the entire population of PC SS. The middle column represents the excitatory PC SS and the right column depicts the population of inhibitory PC SS. Significant changes of the CD during the course of SPA were assessed with Friedman ANOVAs. Excitatory PC SS showed a highly significant increase in CD along the course of gain-increase SPA while inhibitory PC SS exhibited no change during gain-increase SPA. Similarly, excitatory PC SS showed a highly significant decrease in CD during the course of gain-decrease SPA with no changes observed for inhibitory PC SS.

Does the goodness of fit (CD) of individual PC SS change during the course of SPA? Since, velocity was the best kinematic variable explaining the variance of the neural activity, all the following analysis is based on degraded model with velocity as the only kinematic term. Figures 8A,B indicate that PC SS on average exhibited specific changes during the course of SPA, which depended on the type of SPA and the type of pursuit response being excitatory or inhibitory. While excitatory PC SS showed a significant increase in CD during the course of gain-increase SPA (Figure 8A; Friedman ANOVA; *p* < 0.001), they exhibited a significant decrease in CD during the course of gain-decrease SPA (Figure 8B; Friedman ANOVA; *p* < 0.001). On the other hand, inhibitory PC SS did not show any change in CD, neither during gain-increase nor gain-decrease SPA (Figures 8A,B; Friedman ANOVA; *p* > 0.05). The Δ used in Figures 8A,B is 15 ms (note that positive values of Δ indicate that the movement follows changes in firing rate). In summary, only excitatory PC SS improve or compromise their correlation with the kinematic variables during the course of gain-increase and gain-decrease SPA, respectively.

**Figure 9:**
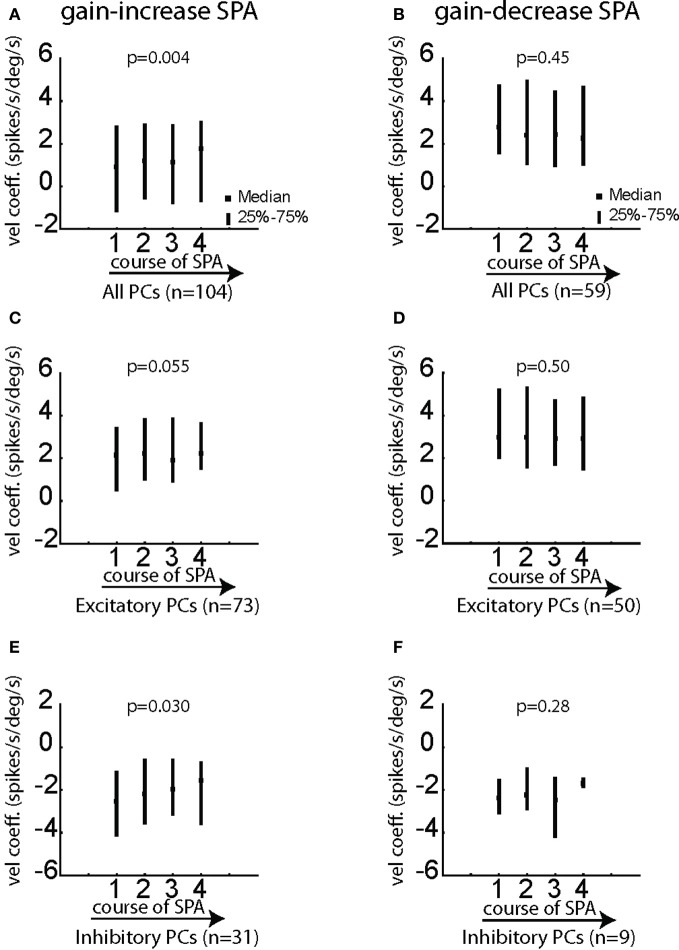
**Changes of the velocity coefficient b in the equation [spk(*t* − Δ) = *f* × vel(*t*) + *h*] for various subgroups of PC SS during gain-increase SPA (A,C,E) and gain-decrease SPA (B,D,F). (A,B)** Show the median/quartile values of the velocity coefficient for the four bins covering the course of SPA for the entire population of PC SS during gain-increase SPA **(A)** and gain-decrease SPA **(B)**. **(C**,**D)** Show the changes of the velocity coefficient b for excitatory PC SS during gain-increase SPA **(C)** and gain-decrease SPA **(D)**. **(E**,**F)** Show the changes of the velocity coefficient for inhibitory PC SS during gain-increase SPA **(E)** and gain-decrease SPA **(F)**. The significance of changes of the velocity coefficient during the course of both forms of SPA was assessed with Friedman ANOVAs (see *p*-values on top of plots). Velocity coefficients in this figure were computed for Δ = 15 ms.

In order to unravel possible changes in the sensitivity to eye velocity of individual PC SS—as shown before the most important kinematic variable—we compared the velocity coefficients yielded by the degraded linear regression model with eye velocity as the only independent variable during the course of SPA. While the mean velocity coefficient for the whole population of PC SS tested for gain-decrease SPA did not show a significant change (Figure 9B; *p* > 0.05; Friedman ANOVA), the mean coefficient of eye velocity exhibited a highly significant increase as a function of adaptation time for the population of PC SS tested for gain-increase SPA (Figure 9A; *p* < 0.005; Friedman ANOVA). We performed the same comparisons for the subpopulations comprising only excitatory or inhibitory PC SS. Excitatory PC SS tested for gain-increase SPA showed a trend toward larger velocity coefficients (Figure 9C; *p* = 0.055; Friedman ANOVA) while inhibitory PC SS exhibited a much stronger increase in velocity coefficients (Figure 9E; *p* < 0.05; Friedman ANOVA). On the other hand, in the case of gain-decrease SPA, the velocity coefficients did not show any change, neither for excitatory nor for inhibitory PC SS (Figures 9D,F; *p* > 0.05; Friedman ANOVA). All the aforementioned results were obtained for Δ = 15.

In summary, PC SS studied during gain-increase SPA and gain-decrease SPA respectively exhibit different changes of their velocity sensitivity. Consistent with the rate analysis shown in Figures 4, 5, changes in eye velocity sensitivity were clear and exhibited by most of the PC SS in the case of gain-increase adaptation, with inhibitory PC SS showing the maximum change. However, no change in velocity sensitivity was observed during gain-decrease SPA.

## Discussion

This study addresses the role of the information conveyed by Purkinje cell simple spikes (PC SS) in the OMV in the adaptation of smooth pursuit eye movements (SPA). We report four major findings: First, OMV PC SS develop changes in their firing rates during the course of gain-increase SPA as well as gain-decrease SPA, both at the level of individual PC SS and at the population level. In general, the direction of the changes depends on the type of adaptation: discharge rate grows during gain-increase SPA and it drops during gain-decrease SPA. However, there was variability in the responses of the individual responses with many PC SS showing adaptation related modulation in the opposite direction. Second, we could replicate our previous finding that the population response—unlike the responses of individual PC SS—can be perfectly predicted by a linear combination of the three kinematic variables characterizing eye movements with eye velocity being the single most relevant variable explaining almost 95% of the discharge variance (Dash et al., 2012). The new finding is that the precise prediction of the population response provided by the linear model holds for any phase of adaptation. Third, while the prediction of the population response based on the linear model was unaltered as a function of adaptation course, at the single PC level the excitatory PC SS showed a significant increase in CD during gain-increase SPA and a significant decrease during gain-decrease SPA. Inhibitory PC SS, on the other hand, did not show any change for either form of SPA. And finally, whereas most of the PC SS demonstrated a profound increase in velocity coefficients during gain-increase SPA, PC SS did not show any change in velocity coefficient during gain-decrease SPA. The increase in velocity coefficients during gain-increase SPA suggests that the gain change occurs at the individual PC level while no change in velocity coefficients during gain-decrease SPA indicates that adaptive gain modulation occurs upstream of the OMV. Although many PC SS showed adaptation related changes in their firing profiles (some also showed opposite changes in SS rate than what would be expected from the direction of SPA), these changes were usually only modestly related to the adaptation induced changes in eye movement kinematics. A much tighter relationship between the discharge and eye movement kinematics as well as their changes due to SPA held for the population signal, which could be predicted practically completely by a linear combination of eye position, velocity, and acceleration, throughout the whole adaptation period. This finding extends observations in a previous study of normal, unadapted smooth pursuit, in which we first demonstrated the perfect prediction of the pursuit-related population response by eye movement kinematics (Dash et al., 2012). The formal structure of the multiple linear regression analysis takes the population signal as the dependent variable determined by eye movement kinematics. However, the clear effects of OMV lesions on SPA suggest that the relationship between discharge and kinematics is actually the reverse of the formal structure of the regression analysis. In other words, we assume that the population signal is responsible for the changes of eye movement kinematics due to SPA. This conclusion is supported by the fact that the best fits were obtained if we assumed that discharge rate changes led changes in eye movements by 15 ms. Very few individual PC SS showed a relationship between eye movement kinematics and their discharge as tight as the one exhibited by the population. This leads to the question if the population signal may simply reflect the contribution of the few “best” PC SS. We think that the answer is no, as averaging contributions of these few “best” PC SS with those of the many more PC SS with very different firing patterns should weaken the relation between discharge and eye movement kinematics, rather than stabilizing or even strengthening it. The alternative, namely, the assumption that PC target neurons in the deep cerebellar nuclei might be able to filter out the signals from the very few “best” PC SS and to ignore all the others, thereby avoiding a deterioration due to averaging, is biologically implausible in view of the structure of the projection from cerebellar cortex to the deep cerebellar nuclei sketched below. In view of these considerations, we rather suggest that the population response is a construct representing more specific information than the underlying components. However, is it also a biologically plausible construct? Why should one assume that the cerebellar control of SPA is based on a population code? Actually, we have argued previously (Thier et al., 2000, 2002; Catz et al., 2008; Dash et al., 2012) that PC SS population coding is a direct consequence of anatomy: we know that many hundreds of PC SS converge and make synapses on single deep cerebellar nucleus neurons (DCN) and, conversely, as many as 35 DCN neurons can be contacted by axon branches of individual PC-units (Palkovits et al., 1977). Actually, a recent study based on physiological recordings suggests a more modest convergence ratio (40:1), i.e., 40 PCs converging on a single DCN neuron (Person and Raman, 2012). Irrespective of the variable estimates of convergence ratios, for a given nuclear neuron, it is impossible to trace a given IPSP back to a particular member in the pool of PC SS contacting this neuron (Bengtsson et al., 2011). Hence, it must be the collective input—in other words—the population signal originating from this pool that determines the state of the nuclear neuron. The notion that a population signal manifested at the level of DCN neurons by averaging the inputs from many individual PCs determines the changes of smooth pursuit based on learning is in full accordance with previous work on short-term saccadic adaptation (STSA) (Catz et al., 2008). The fact that two independent forms of oculomotor learning are based on the same computational principle suggests that PC SS population coding is a generic principle underlying cerebellum-based motor learning at large. How come that the population signal—unlike its constituents—is able to precisely describe the kinematics and their changes during SPA. We have previously argued that the necessary shaping of the PC SS population signals could be achieved by using feedback on the adequacy of the behavior to attenuate and respectively strengthen individual PC SS responses. Our recent study of CSs signals in the OMV during SPA clearly indicates that the climbing fiber system is able to offer information suitable for the modification of the PC SS population response (Dash et al., 2010). In summary, we suggest that mossy fibers offer a rich spectrum of pursuit related discharges, differing both in strength and in duration. Based on feedback reflecting the adequacy of the performance, arguably introduced by the climbing fiber system, inputs are selected—i.e., obtain larger synaptic weights—while others are suppressed. Moreover, the learned changes in the OMV PC population response exclusively terminates on the caudal fastigial neurons (cFN), which in turn has bilateral projections to ventral portions of lateral and inferior vestibular nuclei, contralateral projections to paramedian pontine reticular formation (PPRF), nucleus reticularis tegmenti pontis (NRTP), and superior colliculus, amongst many other areas (Batton et al., 1977; Noda et al., 1990). All the above areas in turn have mono- or di-synaptic connections with the motorneurons. The exact mechanisms of information transfer through these areas with respect to SPEM or SPA are yet to be worked out in detail.

Is the OMV indispensable for SPEM and SPA? Lesions of the OMV impair the initiation of SPEM and SPA, although they do not abolish these oculomotor functions completely. Unlike the effects of OMV lesions on pursuit initiation, the ones on the steady state phase of SPEM are marginal (Takagi et al., 2000). On the other hand, complete cerebellectomy leads to an absolute loss of any SPEM (Westheimer and Blair, 1973, 1974), suggesting additional contributions from non-vermal parts of the cerebellum. The non-vermal regions implicated in pursuit by previous work are the flocculus/ventral paraflocculus and the hemispheric lobuli VI and VII (HVI and HVII) and both have been suggested to also contribute to SPA (Kahlon and Lisberger, 2000; Medina and Lisberger, 2008, 2009; Ohki et al., 2009). With respect to HVI and HVII, this conclusion is based on the observation of a mild deficit in post saccadic pursuit gain and its adaptation following a lesion. As no pertinent single-unit data are available, it remains unclear if the lesion effect reflects a functional contribution to pursuit, qualitatively different from the one of the OMV. With respect to the flocculus/ventral paraflocculus, we are facing the reverse situation. Here, a causal experiment, i.e., a lesion experiment, needed to firmly establish a role of this part of the cerebellum in SPA has never been carried out. On the other hand, recordings from PCs have indeed suggested such a role. Kahlon and Lisberger, using a SPA paradigm very similar to ours, demonstrated changes of the discharge rates in a small sample of flocculus/ventral paraflocculus PC SS (Kahlon and Lisberger, 2000). Later studies by Medina and Lisberger dealt with changes of the discharge patterns of floccular complex PC SS during the learned addition of a directional component 250 ms after target onset (Medina and Lisberger, 2008, 2009). Assuming a normal pursuit latency of 100–150 ms, it is safe to infer that the directional adaptation in their case took place not during pursuit initiation. With respect to the first study, the smallness of the sample of PC as well as differences in the analytic approach such as a lack of a multidimensional kinematic analysis and the absence of information on population responses preclude a direct comparison with the present work. With regard to the second study, a comparison is forestalled by the substantial differences between paradigms. In any case, different roles of the flocculus/ventral paraflocculus and the OMV in SPA are very likely, given the intimate link with the vestibular system in the case of the former and a comparably tight relation to saccades in the case of the latter.

**Figure 10:**
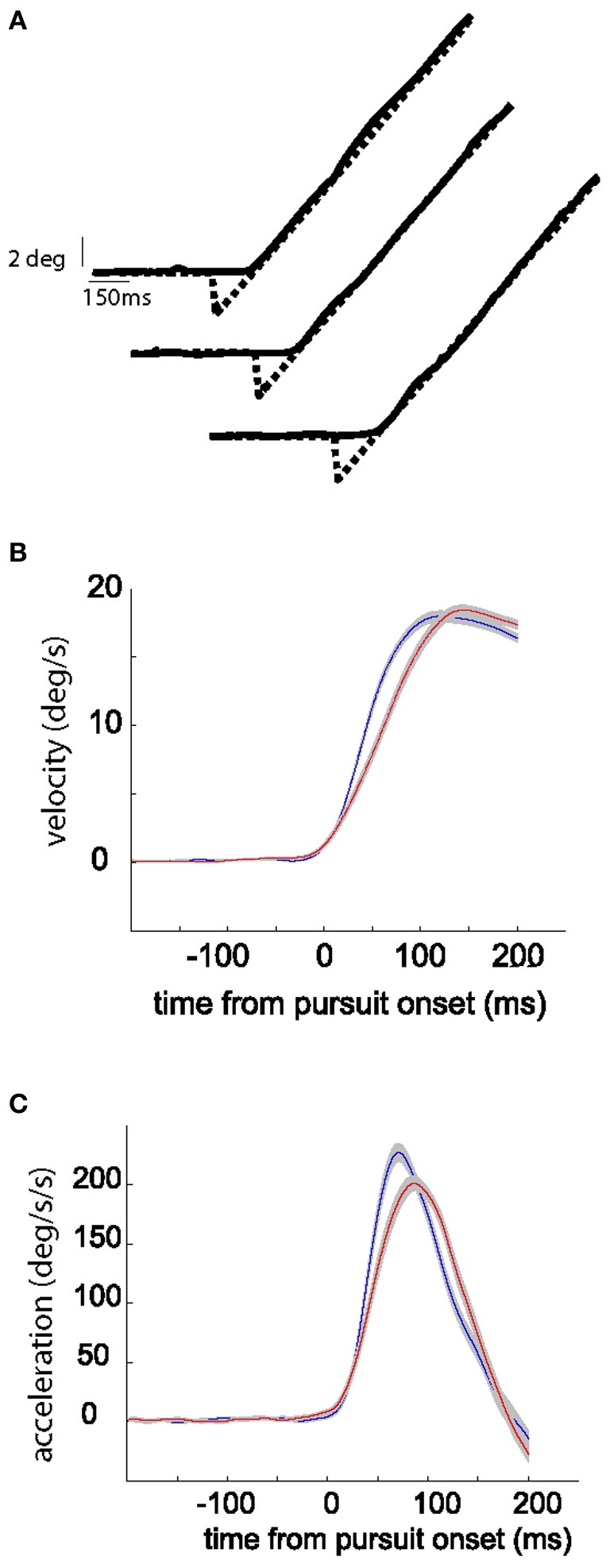
**Top panel shows exemplary target and eye position traces collected in the beginning, in the middle, and at the end of the session during SPEM resilience (A).** Middle panel shows the average eye velocity in the first quarter (blue) and last quarter (red) of trials during a typical SPEM resilience session **(B)**. The lower panel shows the corresponding average eye acceleration traces **(C)**. The gray shadow around the red and blue traces signifies the standard error.

In conclusion, our study adds to the growing list of findings suggesting a role of the OMV in optimizing the behavior of both forms of voluntary eye movements, i.e., saccades and SPEM. OMV micro stimulation evokes both saccades and SPEM (Krauzlis and Miles, 1998) and lesions of the OMV disrupt both types of goal-directed eye movements, and foremost their short-term adaptation based on error information (Takagi et al., 1998, 2000; Barash et al., 1999). Our findings (this work and Catz et al., 2008) suggest that this role of the OMV in saccades and smooth-pursuit, so compellingly demonstrated by these causal experiments, is based on the same computational principle, namely the specification of kinematic parameters as they unfold in time by a PC SS population signal influencing target neurons in the deep cerebellar nuclei. As previous work has provided convincing evidence that at least some individual PCs are responsive to saccades as well as to smooth-pursuit (Suzuki and Keller, 1988), we may assume that overlapping populations of OMV PC SS contribute to both types of goal-directed eye movements. As the kinematic requirements of saccades and smooth-pursuit initiation are not congruent, it seems unlikely that the populations of OMV PC SS accommodating the two types of behavior are fully congruent. This would make it impossible to adjust saccade and pursuit kinematics independently. On the other hand, the fact that there seems to be at least some overlap in the cerebellar machinery for saccades and pursuit clearly emphasizes the existence of important functional commonalities and shared dependencies characterizing saccades and smooth pursuit. We have argued earlier that saccadic adaptation is a manifestation of a brain mechanism subserving the compensation of fatigue (Golla et al., 2008). By the same token, SPA exhibits characteristics, which suggest that SPA may be useful to compensate changes in the properties of the SPEM system due to usage. This conclusion is prompted by a careful comparison of the changes of eye movement kinematics due to gain-decrease and gain-increase SPA on the one hand and the changes exhibited by long series of stereotypic SPEM (resilience) of a constant direction and speed on the other hand (Figure 10A). During a typical pursuit resilience experiment the peak velocity is maintained throughout the session (Figure 10B). However, during the session there is a gradual decline in the peak acceleration, which is compensated by an expansion of the acceleration profile (similar to the expansion of the acceleration profile during gain-increase SPA) (Figure 10C). These adjustments of the acceleration profile are analogous to the changes of the eye velocity profile in the case of saccades: in a saccadic resilience in which a long series of precise stereotypic saccades is required, the peak eye velocity gradually declines accompanied by a fully compensatory increase in saccade duration, maintaining a precise saccade amplitude (Golla et al., 2008). Following a vermal lesion this compensatory increase in saccade duration is compromised (Golla et al., 2008; Xu-Wilson et al., 2009), an observation that supports the notion that an optimized OMV PC SS population response is needed to select the right movement duration (Catz et al., 2008; Prsa and Thier, 2011). Future recording and lesion experiments during SPEM resilience will be needed to test the idea that similarly an optimized OMV PC SS is responsible for the compensation of pursuit fatigue by adjusting the eye acceleration profile.

The population response in our data is potentially contributed by PC SS from both sides of the imaginary OMV midline and that could be a caveat. However, the OMV being a midline structure with no landmark separating left from right, it is difficult to assign laterality to OMV neurons (unlike neurons in many other structures). We identified the OMV based on anatomical MRI and very reliable physiological landmarks. Yet, these criteria do not allow one to reliably distinguish sides. Assigning sides based on the preferred direction of SS responses is not possible as many units lack simple directional preferences. Moreover, we limited ourselves to horizontal pursuit direction and selected the horizontal direction with bigger pursuit related response. One might argue that a careful anatomical reconstruction of recording sites might allow the definition of the side. As the resolution of this method is probably not much better than 1–2 mm and the OMV has a width of only 10 mm a laterality label might only be reliably attached to the few units located in the peripheral zones of the area. However, in unpublished work from our lab on saccade related SS responses, in which we carefully charted the location of units recorded using this approach, we could not find any convincing map of preferred saccade directions. In the case of the present study, we cannot offer an anatomical reconstruction as the animals are still being used for other purposes. Moreover, a number of anatomical arguments favor a distribution of information on both sides of a hypothetical vermal midline: anatomical work has convincingly demonstrated that a given mossy fiber may have terminals on both sides of the midline (Wu et al., 1999). Also parallel fibers, with a length of 3–5 mm, again do not stop at the midline. Finally, also studies of OMV projections to the DCN do not only suggest outflow to the nuclei on one side but also to the other side (e.g., the study by Kralj-Hans et al., 2007). In sum, we think that the anatomy is quite compatible with a distribution of information on both sides of the vermis, although the exact balance between the two sides still needs to be worked out.

### Conflict of interest statement

The authors declare that the research was conducted in the absence of any commercial or financial relationships that could be construed as a potential conflict of interest.
